# The Dynamic Interplay of Healthy Lifestyle Behaviors for Cardiovascular Health

**DOI:** 10.1007/s11883-022-01068-w

**Published:** 2022-11-24

**Authors:** Penny M. Kris-Etherton, Philip A. Sapp, Terrance M. Riley, Kristin M. Davis, Tricia Hart, Olivia Lawler

**Affiliations:** 1 Department of Nutritional Sciences, The Pennsylvania State University, 319 Chandlee Lab, University Park, PA 16802 USA; 2 Department of Biobehavioral Health, The Pennsylvania State University, University Park, PA 16802 USA

**Keywords:** Healthy dietary pattern, Physical activity, Healthy weight, No tobacco, Quality sleep, Stress management, Cardiovascular health

## Abstract

**Purpose of Review:**

The recent rise in cardiovascular disease (CVD) deaths in the USA has sparked interest in identifying and implementing effective strategies to reverse this trend. Healthy lifestyle behaviors (i.e., healthy diet, regular physical activity, achieve and maintain a healthy weight, avoid tobacco exposure, good quality sleep, avoiding and managing stress) are the cornerstone for CVD prevention.

**Recent Findings:**

Achieving all of these behaviors significantly benefits heart health; however, even small changes lower CVD risk. Moreover, there is interplay among healthy lifestyle behaviors where changing one may result in concomitant changes in another behavior. In contrast, the presence of one or more unhealthy lifestyle behaviors may attenuate changing another lifestyle behavior(s) (poor diet, inadequate physical activity, overweight/obesity, poor sleep quality, tobacco exposure, and poor stress management).

**Summary:**

It is important to assess all of these lifestyle behaviors with patients to plan an intervention program that is best positioned for adherence.

## Introduction

For more than 100 years, CVD has been the leading cause of death, and stroke has been the third leading cause of death since 1938 in the USA [1]. While considerable progress was made in decreasing deaths from CVD between approximately 1970 and 2010, there has been a very concerning increase in CVD mortality since 2010 [2]. New strategies are needed to meaningfully improve cardiovascular health. There is strong evidence for the cardiovascular benefits of a healthy lifestyle that includes a healthy diet, physical activity, healthy weight, no tobacco use, quality sleep, and stress management, all of which beneficially affect major risk factors for CVD (dyslipidemia, high blood pressure, and elevated glucose levels). This is the basis for the American Heart Association’s (AHA) Life’s Simple 7 Program [3], recently updated to Life’s Essential 8 Program (to include sleep) which summarizes the modifiable health behaviors and risk factors for cardiovascular health [4].

**Fig. 1:**
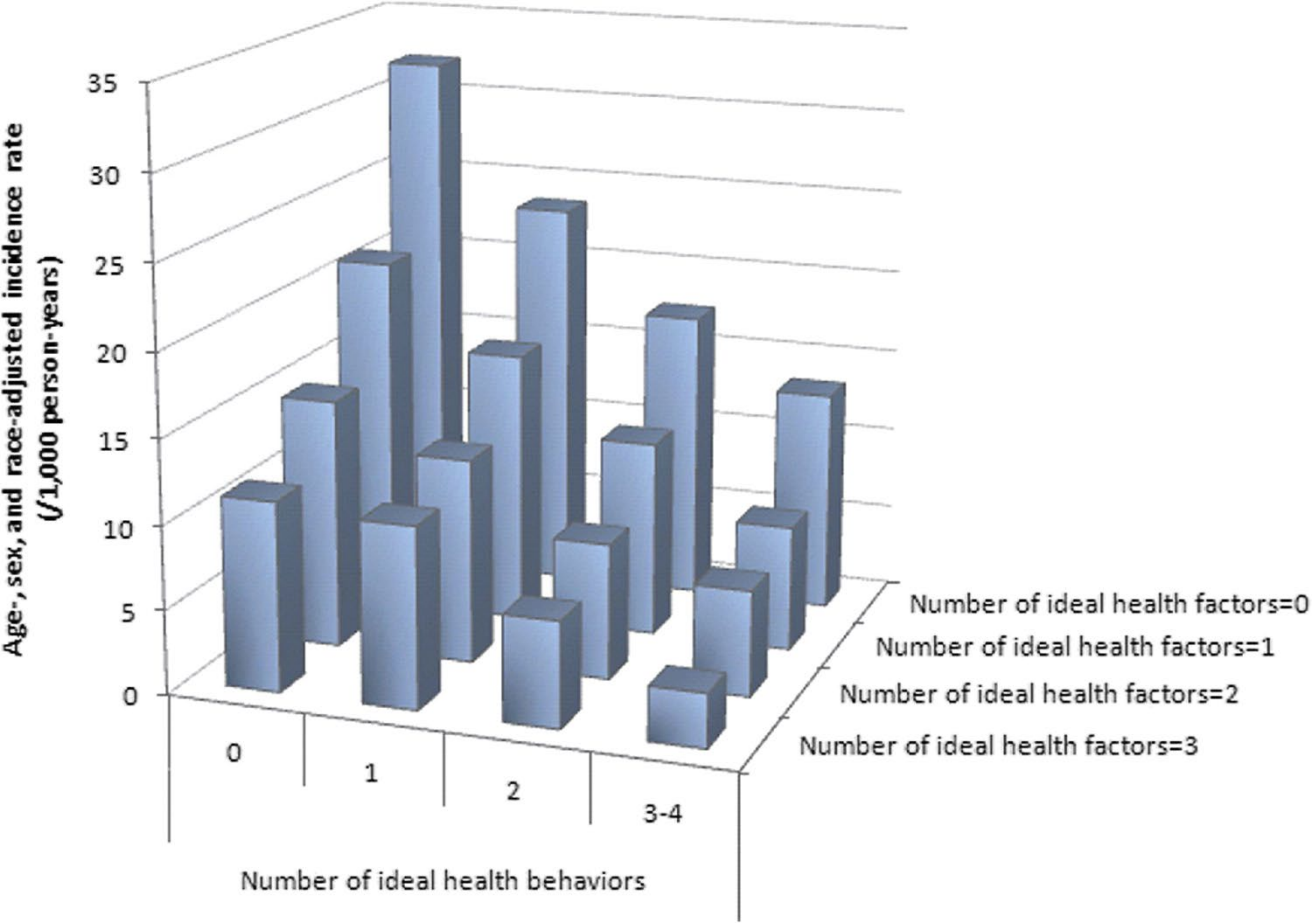
Incidence rate of cardiovascular disease according to the number of ideal health behaviors and health factors. Age-, sex-, and race-adjusted incidence rate of cardiovascular disease according to the number of ideal cardiovascular health behaviors (nonsmoking, body mass index, physical activity, healthy diet score) and health factors (total cholesterol, blood pressure, glucose), ARIC (Atherosclerosis Risk in Communities), 1987 to 2007 (Reprinted from: Folsom et al. 2011 [4], with permission from Elsevier.)

Achieving ideal cardiovascular health markedly decreases incidence of CVD (Fig. 1). For example, the incidence of CVD is linked to the number of optimal health behaviors and health factors, with incident CVD being the lowest when all seven are optimal. Importantly, however, there are benefits of improving just one health behavior (quit/never smoke, achieve a healthy body weight, high diet quality, and physical activity recommendations) or one health factor (i.e., attain normal cholesterol levels, blood pressure, and blood glucose levels) on incident CVD [5]. A recent systematic review and meta-analysis of 20 cohort studies with 1,090,261 participants reported that the group with the highest number of healthy lifestyle practices (nonsmoking, moderate alcohol consumption, healthy diet, physical activity, and optimal weight) versus the group with lowest number had a lower CVD risk [pooled hazard ratio, 0.37 (95% CI 0.31–0.43)] [6]. In agreement, data from the Nurses’ Health Study and the Health Professionals Follow-up Study, demonstrated that 5 low-risk lifestyle behaviors [never smoking, body mass index (BMI) of 18.5 to 24.9 kg/m^2^, ≥ 30 min/d of moderate to vigorous physical activity, moderate alcohol intake, and a high diet quality score (upper 40%)] was associated with multiple health benefits compared to a cohort with zero low-risk behaviors [7]. Implementation of multiple healthy lifestyle behaviors had marked health benefits; the multivariable-adjusted hazard ratios for mortality with 5 compared with zero low-risk factors were 0.26 (95% confidence interval [CI], 0.22–0.31) for all-cause mortality, 0.35 (95% CI, 0.27–0.45) for cancer mortality, and 0.18 (95% CI, 0.12–0.26) for CVD mortality.

Despite convincing evidence of the benefits of healthy lifestyle behaviors, the vast majority of Americans do not meet ideal criteria for components of cardiovascular health except for avoiding tobacco use, i.e., smoking (77.6–91.6% meet smoking criteria for ≥ 20 years of age, 2017–2018). Most adults (≥ 20 years of age) across all racial groups fall short of ideal criteria for BMI (14.2–44.7%), physical activity (29.6–40.1%), blood pressure (31.0–43.2%), and healthy diet score (0.0–1.5%), whereas total cholesterol is ideal for slightly more than half of adults (50.1–58.3%) [2]. Consequently, there is a pressing need to identify strategies that promote the adoption of healthy lifestyle behaviors. Using the 5A model (assess, advise, agree, assist, and arrange) for behavior change, two recent AHA Scientific Statements have provided the framework for counseling on behavior change to achieve healthy lifestyle behaviors [8•, 9•].

**Fig. 2:**
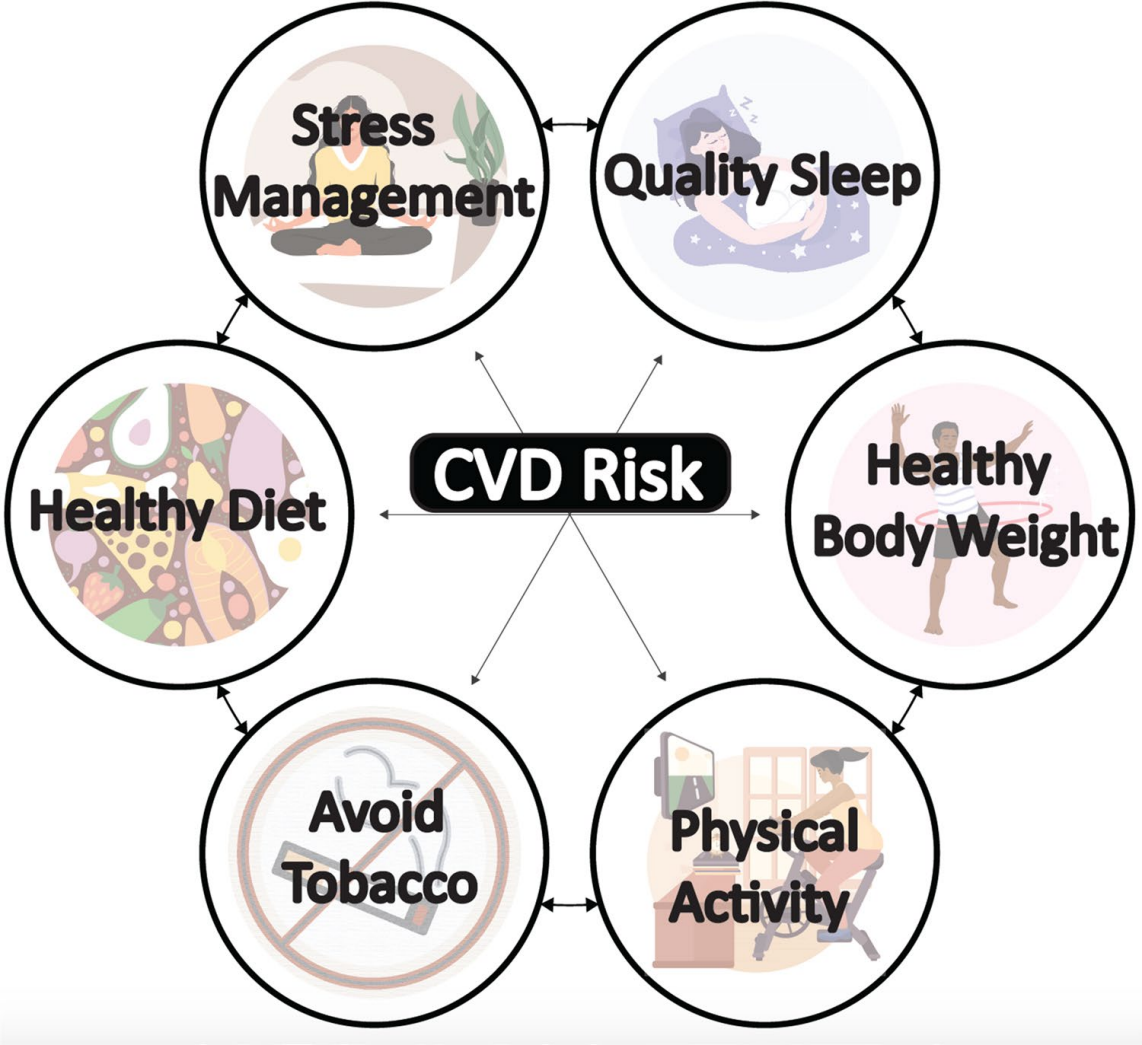
The dynamic interplay of lifestyle behaviors that affect CVD risk (all images obtained from free Adobe Stock Images (Extended License). Modifications performed by TMR. Attributions: Sleep image—Artinspring (#313,612,892), Sophie Alp—(#454,458,241), Stress Management—Cвeтлaнa Бapминa (#413,814,883), Avoid Tobacco – Sir.Vector (#482,645,816), Physical Activity – freeslab (#436,440,860), Healthy Diet – Maria (#304,907,052)

The evidence for the CVD benefits of changing individual healthy lifestyle behaviors is robust. Moreover, there is some evidence that changing one lifestyle behavior benefits others. This interplay is depicted in Fig. 2. The effect of targeting just one healthy lifestyle behavior and the interaction with other healthy lifestyle behaviors is less discussed. The purpose of this paper is to briefly review the benefits of a healthy diet, physical activity, healthy weight, no tobacco use, quality of sleep, and stress management individually on CVD risk based on cohort studies and intervention studies and how changing each of these behaviors may impact other healthy lifestyle behaviors. An awareness of the dynamic interplay among healthy lifestyle behaviors will help identify lifestyle intervention strategies to improve cardiovascular health.

## A Healthy Dietary Pattern

Evidence from epidemiologic studies and clinical trials has shown that a healthy dietary pattern significantly reduces risk of CVD and also major risk factors for CVD. Strong and consistent evidence from epidemiologic studies has shown that higher diet quality is associated with lower relative and absolute risk of CVD and also longer CVD-free survival time [10]. In the Nurses’ Health Study I and II and the Health Professionals Follow-up Study, participants in the highest diet quality quintile compared with the lowest diet quality quintile had a 14–21% lower risk of CVD [11]. Likewise, in the Women’s Health Initiative Observational Study, a higher diet quality was associated with an 18–26% lower risk of all-cause and CVD mortality [12]. There is convincing clinical trial evidence demonstrating that a healthy dietary pattern decreases multiple major CVD risk factors, including elevated LDL-C and blood pressure [13] in addition to other cardiometabolic risk factors (i.e., increased blood glucose, triglycerides, waist circumference, and body weight) [14]. Based on these collective findings, the 2020 DGAC Committee concluded that there was strong and consistent evidence that a healthy dietary pattern is associated with a decreased risk of CVD [13], a conclusion that aligns with the 2021 AHA Scientific Statement, Dietary Guidance to Improve Cardiovascular Health [15•].

It is well known that social and behavioral risk markers (e.g., physical activity, diet, smoking, and socioeconomic position) cluster and have a corresponding impact on risk of CVD [16, 17]. With respect to social determinants, the AHA Healthy Diet Score is related to race and ethnicity, and to the family income to poverty ratio, such that diet quality decreases with lower economic status [2]. In addition, in the Women’s Health Initiative, women reporting a higher diet quality at baseline were non-Hispanic white, educated, physically active, past or never smokers, hormone therapy users, had lower BMI and waist circumference, and were less likely to have chronic conditions [18]. Evidence of a healthy diet clustering with other healthy lifestyle behaviors comes from a cross-sectional analysis of the Prevención con Dieta Mediterránea (PREDIMED)-Plus Trial designed to evaluate the effects of a Mediterranean Diet plus body mass reduction achieved by physical activity promotion and dietary energy reduction in 6646 participants living in Spain [19]. Lifestyle factors including nonsmoking and avoidance of sedentary lifestyles were associated with better diet quality. Moreover, in a cross-sectional analysis conducted with participants in the PREDIMED Trial, better sleep quality was related to higher adherence to a Mediterranean Diet, a lower BMI and waist circumference compared to poor sleep quality [20].

Given that a healthy diet clusters with other lifestyle behaviors, questions persist about whether a diet intervention specifically can “spillover” and benefit other lifestyle behaviors and, in fact, be a gateway for a change in other lifestyle behaviors not targeted. In a short literature review on this topic, Sarma et al. concluded that the evidence is inconsistent, which prompted their study to evaluate the effect of a diet intervention on other lifestyle modifications in participants in the Women’s Health Initiative (WHI) [21]. After 1 year, there was no change in physical activity, alcohol consumption, and smoking behavior in women in the diet intervention group leading the authors to conclude that diet modification does not have a spillover effect on untargeted behaviors and that changing multiple health behaviors may require targeting additional behaviors. However, there were benefits of the dietary intervention on several health factors and behaviors in WHI. For example, the dietary intervention benefited body fat percentage (− 0.8% [95% CI − 1.0%, − 0.6%]) despite this not being targeted for change [22]. Moreover, a 10% increase in diet quality was associated with a smaller increase in waist circumference (0.07 to 0.43 cm) after 3 years (all *P* < 0.05) [23]. In addition, at 1-year follow-up, the diet intervention was associated with significant improvements in three health-related quality of life (HRQoL) subscales: general health, physical functioning, and vitality [24]. Finally, there is evidence that improvements in diet quality scores are related to optimism and good mental health in postmenopausal women [18, 25].

## Physical Activity

Current recommendations for physical activity are 150 min per week of moderate physical activity or 75 min per week of vigorous physical activity, which is equivalent to 11.25 metabolic equivalent hours per week (MET h/week) [26, 27]. Based on an extensive literature review of physical activity and health issued by the 2018 Physical Activity Guidelines Advisory Committee, there is a strong evidence for an inverse dose–response relation between moderate-to-vigorous physical activity and CVD incidence [26, 28]. Adults increasing physical activity from baseline inactivity to low (0–11.5 MET h/week), medium (11.5–29.5 MET h/week), and high (> 29.5 MET h/week) physical activity had a reduced risk of CVD incidence (0.89 [95% CI 0.82, 0.98]; 0.79 [95% CI 0.69, 0.89]; and 0.75 [95% CI 0.64, 0.87]) [26]. In addition, there is strong evidence demonstrating a significant relationship between greater amounts of physical activity and decreased incidence of CVD, stroke, and heart failure. Physical activity also decreases CVD risk factors, including overweight or obesity, hypertension, high blood cholesterol, and blood glucose [29, 30].

A meta-analysis of 33 studies evaluating the relationship between CVD and physical activity with an average follow-up of 12.8 years found that sedentary adults (ages 25–93 years) that increased physical activity to the current recommendation (11.25 MET h/week) had a 23% (95% CI, 0.71–0.84) reduction in risk of CVD mortality and a 17% (95% CI, 0.77–0.89) reduction in CVD incidence [26]. Additionally, individuals who were inactive (0 MET h/week) and incorporated even small amounts (6 MET h/week) of physical activity into their routine had a reduction in CVD risk [26]. This increase in sedentary to light activity over a ~ 13-year period resulted in a risk reduction of 4.3% per MET h/week for CVD mortality and 1.7% for CVD incidence [26]. Potential mechanisms for the dose-dependent response of exercise to decreased CVD risk include improved glycemic control [31], improved vascular health [29, 32, 33], and improved leptin sensitivity [34].

While the focus of physical activity attenuating CVD risk is often associated with concurrent weight loss, physical activity benefits CVD risk independent of body weight changes [26]. An AHA meta-analysis highlighted several trials showing physical activity, independent of weight loss, benefits CVD mortality with only moderate changes in risk ratios when adjusting for weight loss [26, 35]. Moreover, a NHANES study (2007–2016) with 22,476 adults revealed a lower 10-year CVD risk when comparing obese inactive (1–149 min/week moderate to vigorous activities) (OR: 0.66 [95% CI: 0.49, 0.89]) and obese active (≥ 150 min/week moderate to vigorous activities) (OR: 0.50 [95% CI: 0.37, 0.69]) to obese and sedentary individuals (0 min/week moderate to vigorous activities) [36]. Across all BMI categories, active individuals had a lower 10-year CVD risk than inactive adults (< 150 min/week) [36]. Although physical activity independently improves CVD risk, the addition of other healthy lifestyle factors provides cumulative benefits [16]. When nutrition education alone is compared with education plus physical activity, the combined intervention resulted in greater weight loss (10.9 kg [95% CI 9.1–12.7]) than nutrition education over 6 months (8.2 kg [95% CI 6.4–9.9]) [37].

Meeting physical activity recommendations may influence other lifestyle behaviors. The US Department of Health and Human Services Advisory Committee reported that regular physical activity could improve sleep, reduce anxiety, slow or reduce weight gain, prevent weight regain after initial loss, and contribute to weight loss [30]. The 2018 Physical Activity Guidelines Advisory Committee Scientific Report rated the evidence as strong for the effect of moderate-to-vigorous exercise on improvements in sleep quality, reducing both acute and chronic anxiety and preventing obesity [38]. Thus, increasing physical activity can benefit other lifestyle behaviors to lower CVD risk.

## Healthy Weight

Clinical and observational studies support a consistent relationship between body weight, CVD risk, and CVD-related risk factors. Weight status is often classified by a BMI score calculated by kg/m^2^ (underweight, ≤ 18.5; normal weight, 18.5–24.9; overweight, 25–29.9; obesity, 30–34.9; and morbid obesity, ≥ 35). A recent review of meta-analyses of observational studies reported a consistent positive association between BMI and CVD risk [39]. Khan et al. assessed the relationship between CVD and BMI with data from 10 US cohorts (3.2 million person-years) and reported that, compared to men and women with a normal BMI, those with an overweight, obese, and morbidly obese BMI had a 21–32%, 67–85%, and 253–314% increased risk of having a CVD event [40]. The long-term effects of weight loss on CVD outcomes are limited, but there is substantial clinical trial evidence demonstrating that weight loss improves dyslipidemia [41], blood pressure [42], and glucose [43]. Based on epidemiological and clinical trial evidence, a recent AHA Scientific Statement on Obesity and CVD concluded that obesity contributes to increased CVD risk factors and the development of CVD. Further long-term clinical trials assessing lifestyle interventions for weight loss are necessary [44].

Body weight (BMI) and healthy lifestyle behaviors (e.g., diet, physical activity, stress, and sleep) are interconnected. In an analysis of the National Health and Nutrition Examination Survey (NHANES) III data, women and men, respectively, had 8.3 and 14.5% lower odds of abdominal obesity for each 10-point increase in Healthy Eating Index (range: 0–100) [45]. Ford et al. analyzed data from the Geisinger Rural Aging Study and reported that health and activity limitation index (perceived health and activity limitation) scores were significantly higher for adults with a normal BMI compared to those with an underweight, overweight, or obese BMI [46]. Similarly, in a cross-sectional analysis of UK adults, greater weekly physical activity was inversely associated with BMI and body fat percentage [47]. Lastly, cross-sectional studies consistently show an inverse association between BMI and sleep quality, but further research is necessary to understand this association and determine causation [48]. There is a consistent relationship between BMI and other lifestyle behaviors, but it is important to understand how these behaviors change when body weight, or BMI, improves.

There is consistent clinical evidence supporting dietary modification and increased physical activity for weight loss [49], but few trials have evaluated the effects of weight loss on other lifestyle behaviors. Das et al. conducted a 12-month parallel randomized controlled trial in free-living adults comparing two weight-loss interventions with the common goal of reducing calories (500–1000 kcal/day) and achieving physical activity goals (150 min/week), but one emphasized weight loss through tracking food and physical activity (modified Diabetes Prevention Program [m-DPP]) and the other emphasized behavior change (stress management, mindful eating, etc.) (Healthy Weight for Living [HWL]) [50]. Both interventions (m-DPP and HWL) significantly improved weight (− 7.32 and 7.46 kg) and cardiometabolic risk factors (LDL-C [− 10 and − 15 mg/dL], triglycerides [18 and − 21 mg/dL], and glucose [− 3 and − 6 mg/dL]) from baseline with similar non-significant improvements in sleep, emotional, and general health. Additionally, a systematic review of studies on behavioral and dietary weight loss interventions found simultaneous improvements in depressive symptoms, body image, and health-related quality of life (perceived physical and mental health) with weight loss [51].

## Avoid Tobacco Exposure

Tobacco use is a well-established, major risk factor for CVD. Components in tobacco products are causally linked to diseases of every major organ system mediated in part by dysfunction of the heart and vasculature [52–54]. Although smoking prevalence has declined in the USA over the last several decades, tobacco smoking is the second leading risk factor of overall disease burden [55] and second leading population attributable fraction (PAF: 13.7% [95% CI 4.8–22.3%]) for CVD mortality [56]. A retrospective analysis of 5 cohorts (*n* = 2.2 million) from 2000 to 2010 showed a 3 times greater risk of death from ischemic heart disease (IHD) in smokers aged 55–74 compared to never smokers [57]. The risk of IHD increases in a dose-dependent manner with the number of cigarettes smoked per day. The Pooling Project on Diet and Coronary Heart Disease (CHD) comprising 12 prospective cohorts (*n* = 266,787) showed that the probability of CHD in smokers, relative to never smokers, is the highest in women aged 40 to 49 years (HR: 8.5 [95% CI 5.0–14.0]) and that the majority of CHD cases were attributable to smoking among all age groups (40 to 49 years, 88% (95% CI = 82%, 94%); 50 to 59 years 81% (95% CI = 77%, 85%); 60 to 69 years, 71% (95% CI = 65%, 76%), + 70 years, 68 (95% CI = 53%, 82%)) [58].

Tobacco use in the form of electronic nicotine delivery systems (i.e., electronic(e)-cigarettes, vaping, etc.) is also of significant concern. E-cigarette use has increased over the last decade primarily in adolescents (12–19 years of age) [59]. In 2019, the National Youth and Tobacco Survey found that about 8 million (53.3%) high school students and 2.9 million (24.3%) middle school students reported ever using tobacco products of which e-cigarettes were the most common (35.0%) [9•]. Although the long-term effects are not yet confirmed, the metabolic damage seen in e-cigarette users aligns with those seen in smokers. Current evidence suggests e-cigarettes and vaping results in systemic inflammation, endothelial dysfunction, vascular stiffening, and increased blood pressure [59]. Due to the similarities in metabolic effects, use of electronic nicotine delivery systems is likely to cause adverse CVD outcomes.

Cessation of tobacco use independently and in combination with CVD-related health behaviors (e.g. diet, exercise, weight management) improves CVD risk. A meta-analysis of 25 cohorts (*n* = 503,905) of men and women 60 years and older showed lower cardiovascular mortality risk for former smokers (HR: 1.37 [95% CI 1.25–1.49]) than current smokers (2.07 [95% CI 1.82–2.36]) when compared with non-smokers over an 8 to 13 year follow up [60]. Early cessation improves health outcomes such that quitting smoking between 25 and 35 years of age is associated with up to a 10 year longer life expectancy [57]. The benefits may be augmented when accompanied by healthy behaviors. In a cohort (*n* = 32,887) of Chinese adults with prehypertension (SBP, 120–139 mm Hg; DBP, 80–89 mm Hg), there was a stepwise risk reduction (~ 14%) of progression to hypertension for each increase in the number of health behaviors (0 to > 5), including quitting smoking, over 6 years [61]. The Organization to Assess Strategies in Acute Ischemic Syndromes (OASIS) Registry of five trials including 18,809 patients from 41 countries with unstable angina or myocardial infarction demonstrated that smoking cessation coupled with diet and exercise improved the odds of a repeat CVD event (OR: 1.62 [95% CI 0.96–2.75]) compared to persistent smoking without modification of diet or exercise (OR: 3.77 [95% CI 2.40–5.91]) over 6 months [62]. Comparatively, persistent smokers without diet and lifestyle changes had 3.77 higher odds (95% CI: 2.40–5.91) of a repeat event. These findings support the incorporation of treatments that target healthy behaviors in addition to smoking cessation to improve CVD-related outcomes.

Tobacco cessation interventions have been shown to adversely affect healthy lifestyle behaviors and CVD risk factors. Increases in caloric intake, appetite, and weight following smoking cessation have been commonly reported and may reduce abstinence [63]. A recent Cochrane systematic review and meta-analysis of 83 trials examining intervention designed to aid smoking cessation (e.g., diet, exercise, nicotine replacement therapies) found that exercise reduced weight gain (mean difference − 2.07 kg [95% CI − 3.78 to − 0.36]) at 12 months but not immediately following the interventions [64]. Mean difference in personalized weight management support (i.e., very low-calorie diet, diet education) showed no differences in weight reduction. However, many results were of low certainty of evidence suggesting that more research combining cessation interventions with other healthy behaviors is needed. Nonetheless, since 68% of smokers desire to quit [65], integrating cessation interventions with other healthy lifestyle behaviors will be most beneficial to CVD risk.

Addressing negative impacts to healthy behaviors with smoking cessation may require an environmental and social approach. A recent rapid-realist review (*n* = 138 trials) using a behavior change framework (motivation, capability, opportunity) suggests that the best approaches for successful smoking cessation in public health promotion interventions should shift from “individualistic epidemiology” to targeting of external factors [66•]. Studies that improve resource access (i.e. healthy foods, exercise), changed the physical environment (smoke-free policies), and improved support networks (including family members into care, social events) were more likely to help with cessation. Moreover, interventions that modified external factors intended to prompt or facilitate multiple healthy behaviors were associated with greater success, whereas successful interventions with an individualistic focus (i.e., enhancing knowledge/skills) tended to be context dependent. Overall, innovative clinical and public health approaches combining smoking cessation and environmental factors as parts of multicomponent behavioral interventions are needed to determine effective strategies in health promotion.

## Sleep Quality

Sleep is considered to be an emerging risk factor for CVD [67]. There is growing evidence that both sleep duration and sleep quality affect CVD risk development [68]. In the Multi-Ethnic Study of Atherosclerosis (MESA) with 1992 participants free of CVD, those with most irregular sleep duration or timing versus those with the most regular sleep patterns had more than a twofold greater risk of developing CVD after 4.9 years of follow-up [69]. A large prospective cohort study of 60,586 adults in Taiwan examined the impact of sleep quality and duration on the risk of CHD and reported that short sleep duration (< 6 h/day) and poor sleep quality (measured by sleep index score—comparing the lowest versus the highest quintiles) increased risk of CHD by about 30% [70]. Similarly, in an analysis of 407,500 participants in the UK Biobank Study, excess sleep (over 9 h) or short sleep (under 5 h), compared to the reference group (7 h), was associated with a higher risk of CVD mortality (HR: 1.27 [95% CI 1.09–1.49] and HR: 1.32 [95% CI 1.16–1.50], respectively) and CVD incidence (HR: 1.23 [95% CI 1.16–1.31] and HR: 1.08 [95% CI 1.02–1.15], respectively) [71]. Sleep irregularity also has been shown to increase cardiometabolic disease risk. In a review of cross-sectional and prospective studies published between 2015 and 2020, Zuraikat et al. reported that greater variability in sleep duration and sleep onset was associated with increased risk for metabolic syndrome, type-2 diabetes, and obesity [72].

Studies of coronary patients also have shown an association between sleep duration and increased risk of CVD. Using data from the Emory Cardiovascular Biobank of 2846 patients with coronary artery disease, both short sleep duration (< 6.5 h) and long sleep duration (≥ 7.5 h) compared to normal sleep (≥ 6.5 and < 7.5 h) were associated with higher all-cause mortality (HR: 1.44 [95% CI 1.10–1.89] and 1.41 [95% CI 1.08–1.85], respectively) [73]. For cardiovascular mortality, only short sleep duration increased risk (HR: 1.48 [95% CI 1.05–2.09]) [73]. In the Progression of Early Subclinical Atherosclerosis Study of 3974 patients, those who slept less than 6 h had a greater preclinical atherosclerotic burden versus the reference group (who slept 7–8 h) [74].

Poor quality sleep adversely affects diet quality and physical activity, which could, in part, explain the cardiometabolic health consequences related to unhealthy sleep. In the AHA Go Red for Women prospective cohort study of 495 participants, poor sleep quality was associated with greater food intake and lower-quality diet (i.e., lower unsaturated fat intake; higher food weight, energy, and added sugars consumed; and a lower intake of whole grains) [75]. Similarly, a review on sleep, diet, and cardiovascular health by St-Onge and Zuraikat suggests sleep restriction increases unhealthy food choices and energy intake [76]. Short sleepers (< 7 h) reported lower intakes of fruits and vegetables and fiber, and higher intakes of saturated fat and snacks relative to adequate sleepers (at least 7 h). In a meta-analysis of 11 randomized controlled sleep restriction studies, the average increase in energy intake was 385 cal per day after partial sleep deprivation (3.75 to 5.5 h for several days) compared to adequate sleep (7–9 h) [77]. Interestingly, there also is a relationship between poor diet quality and poor sleep quality. In a systematic review of nine studies that assessed the relationship between diet quality and sleep quality, consumption of a healthy dietary pattern (which included high intake of plant derived foods, whole grains, legumes, seafood, and olive oil) was associated with better sleep quality, whereas higher intake of processed foods and added sugars was associated with lower sleep quality [78].

Poor sleep quality affects other lifestyle behaviors, including physical activity and stress-related disorders. A systematic review and meta-analysis including observational and intervention studies examined daily associations between sleep and physical activity and reported that three sleep parameters (sleep quality, sleep efficiency, and wake after sleep onset) were associated with physical activity the following day [79]. The authors noted, however, that the associations were small and variable relative to the sleep parameter assessed (sleep quality, sleep efficiency, and wake after sleep onset) that could have been related to the different methods used to evaluate both sleep measures and physical activity. In addition, sleep and physical activity associations were evaluated in ten studies, four of which associated high sleep quality with higher total physical activity, whereas six studies reported no association. In the UK Biobank cohort of 82,995 participants, an inverse “U-shaped” relationship between objective sleep duration and physical activity level identified the most physical activity reported for those who slept between 6 and 7 h [80]. Another recent meta-analysis reported that sleep disturbances increase risk of anxiety-related disorders [81] with some evidence indicating that sleep deprivation adversely affects stress.

Collectively, the evidence shows many associations between sleep quality and risk of cardiovascular/cardiometabolic diseases. The associations of sleep with other lifestyle behaviors including diet quality, physical activity, and stress management reinforce the importance of getting quality sleep for heart health. However, evidence suggests that poor adherence to other lifestyle behaviors (e.g., poor quality diet) can adversely affect sleep quality, which underscores the importance of understanding of how all lifestyle behaviors interact to affect cardiovascular health.

## Stress Management

The association between psychosocial stress and CVD risk has substantial empirical support [82–85]. Prospective observational studies have found that chronic stress is associated with a 40–50% increase in CHD risk [82]. Results from the INTERHEART study suggest that general stress increases risk of myocardial infarction by 38% (several periods of stress) to 114% (permanent stress) [86]. Stress has also been associated with increased risk of other forms of CVD, including stroke and atrial fibrillation [87], hypertension, blood pressure fluctuation, and carotid artery plaque [88], and stress (Takotsubo) cardiomyopathy [89]. Briefly, acute psychosocial stress activates two key stress response systems, the sympathetic nervous system (SNS) and the hypothalamic–pituitary–adrenal (HPA) axis. Activation of the SNS results in increased inflammation, potentially accelerating the atherosclerotic process [90]. The end product of the HPA axis, cortisol, has anti-inflammatory properties; however, under conditions of chronic stress, glucocorticoid resistance can result in upregulated inflammation [91, 92]. Between-person differences in the degree of SNS and HPA axis activation may moderate the association between perceived stress and CVD risk [83]. Similarly, augmented activity of the amygdala has been implicated in the association between stress and CVD risk; because the amygdala is a brain region that regulates physiological stress responses, increased activity here implies greater stress reactivity [83]. Chronic or traumatic stress in childhood compared to adulthood appears to have a stronger effect on CVD risk [87]. However, stress in adulthood can accelerate the development of atherosclerosis, act as a disease trigger in individuals with advanced atherosclerotic plaques, and adversely affect CVD prognosis and outcomes [87, 93]. Cultivating healthy stress management practices has shown promise for both the primary and secondary prevention of CVD [94] leading to the inclusion of stress management in some clinical guidelines for CVD risk reduction [95].

Stress is often tied to the development of maladaptive coping mechanisms, with a negative impact on CVD outcomes [82, 83]. High stress levels are associated with poor health behaviors, including worse diet quality [96–101]; less physical activity [85, 101, 102]; increased use of tobacco, alcohol, and other drugs [85, 103–106]; and reduced sleep quantity and quality [107–110]. Stress also negatively impacts body weight and body composition [96, 111]. Those experiencing ≥ 3 chronic stressors have a 50% increase in odds of obesity and are more likely to have an elevated waist circumference and body fat percentage compared to those without chronic stress [96]. Poor health behaviors mediate the association between stress and CVD risk, accounting for 65% of the observed variance [85]. Evidence suggests that the relationship between health behaviors and stress is often bi-directional. For example, those with high stress levels are less likely to engage in regular physical activity [101, 102], and regular exercise has been shown to improve stress reactivity [112, 113] and reduce perceived stress [114]. Similarly, while high stress levels are often associated with sleep impairments, sufficient sleep quantity and quality can reduce stress levels [107, 108, 115]. This bi-directionality has potentially wide-reaching benefits for prevention and intervention research: targeting stress may help improve other health behaviors, and targeting other health behaviors may be an effective way to improve stress management.

Stress management- and stress reduction-based intervention techniques are widely used, especially in the context of improving health behaviors [116–118]. Mindfulness-based interventions appear to be a particularly valuable tool for improving stress management abilities, with positive impacts on CVD risk [119]. To date, few interventions using stress reduction to improve diet quality have been tested, but initial results are promising. For example, mindfulness-based stress reduction interventions have successfully reduced sweets consumption in obese adults [120] and improved diet quality in healthy adults [121]. Research is needed to investigate the impact of stress reduction on physical activity. Results thus far have been conflicting: some studies have found that stress reduction improves physical activity outcomes, others have reported no association, and still others have observed adverse outcomes [102]. Evidence suggests that weight loss interventions are less effective for highly stressed individuals; very few intervention studies have targeted stress management as a weight loss intervention [111]. Of two known studies, both are small (*n* = 34 and 44), and only one reported that including stress management significantly improved weight loss [122]. The other reported a non-significant trend toward improved weight loss with the addition of stress management [123]. Use of stress management as a tobacco cessation tool is well-supported. Evidence suggests that tobacco is often used to cope with stress, and that smoking cessation requires the development of healthier coping strategies [124]. Stress management interventions reduce tobacco use [125] and improve cessation outcomes, including reducing the risk of relapse [126, 127]. Meta-analyses suggest that mindfulness-based stress reduction techniques may help improve sleep in individuals with insomnia [128] and in individuals without sleep disorders [118], but mixed results have been reported [129]. One interesting longitudinal study found that employees given more control over their work schedules reported less stress and getting an extra 30 min of sleep on weeknights [130]. Thus, stress management is a promising avenue for improving other health behaviors, including diet, physical activity, tobacco use, and sleep.

## Conclusion

It is important for clinicians to be aware of the interplay of lifestyle behaviors. Modifying just one lifestyle behavior may be hindered by the presence of other unhealthy lifestyle behaviors. Therefore, identifying underlying lifestyle behavior-related problems (such as poor sleep, high stress) that could prevent the modification of a selected behavior will help guide a successful lifestyle behavior intervention program to improve heart health. Identifying a lifestyle behavior to target initially requires shared decision-making between the clinician and patient with the goal being to help the patient modify the selected lifestyle behavior(s). For some patients, targeting multiple lifestyle behaviors simultaneously may work. Regardless, it is important to be mindful of the status of all of the patient’s lifestyle behaviors in order to implement interventions that achieve optimal cardiovascular health.

Further research is needed to better understand the dynamic interplay of healthy lifestyle behaviors for cardiovascular health. Current lifestyle behavior research often focuses on just one specific lifestyle behavior. Future clinical trials need to evaluate the interplay of the multiple lifestyle behaviors discussed herein with the objective being to identify the best intervention strategies for successful lifestyle behavior change for improved cardiovascular health.
